# Infectious complications following transperineal prostate biopsy with or without periprocedural antibiotic prophylaxis—a systematic review including meta-analysis of all comparative studies

**DOI:** 10.1038/s41391-024-00934-9

**Published:** 2024-12-31

**Authors:** Ingmar Wolff, Markus Büchner, Katharina Hauner, Florian Wagenlehner, Martin Burchardt, Marianne Abele-Horn, Bernd Wullich, Christian Gilfrich, Adrian Pilatz, Matthias May

**Affiliations:** 1 Department of Urology, University Medicine Greifswald, Greifswald, Germany; 2 Department of Urology, School of Medicine and Health, TUM University Hospital, Technical University of Munich, Munich, Germany; 3 Department of Urology, Pediatric Urology and Andrology, Justus Liebig University Giessen, Giessen, Germany; 4 Institute for Hygiene and Microbiology, University of Würzburg, Würzburg, Germany; 5 Department of Urology and Pediatric Urology, University Hospital Erlangen, Friedrich-Alexander Universität Erlangen-Nürnberg, Erlangen, Germany; 6 Department of Urology, St. Elisabeth Hospital Straubing, Brothers of Mercy Hospital, Straubing, Germany

**Keywords:** Prostate cancer, Cancer screening

## Abstract

**Background:**

Despite the relatively low infection rate following transperineal prostate biopsy (TPB), it remains unresolved whether periprocedural antibiotic prophylaxis (PAP) can be omitted. Our aim was to compare infectious complications (genitourinary infections/GUI, fever, sepsis, readmission rate, 30-day-mortality) following TPB, considering all studies of varying levels of evidence that enable a direct comparison between patients with and without PAP.

**Methods:**

We performed a comprehensive search in PubMed/Medline, Embase, Web of Science, and Cochrane databases, as well as grey literature sources, to identify reports published until January 2024. All studies comparing the incidence of infectious endpoints following TPB with vs. without PAP were included in the analyses. The GRADE approach was employed to assess the certainty of evidence for each comparison.

**Results:**

Twenty-three studies met the inclusion criteria involving 6520 and 5804 patients who underwent TPB with vs. without PAP, respectively. Two of the 23 studies were randomized-controlled trials, not all studies investigated all endpoints. Pooled incidences between patients with vs. without PAP for the endpoints GUI (0.50% vs. 0.37%), fever (0.44% vs. 0.26%), sepsis (0.16% vs. 0.13%), and readmission rate (0.35% vs. 0.29%) showed no significant differences (all *p* > 0.250). The corresponding odds ratios (including 95% confidence interval) also revealed no statistically significant differences: 1.37 (0.74–2.54) [GUI], 0.87 (0.28–2.66) [fever], 1.30 (0.46–3.67) [sepsis], and 1.45 (0.70–3.03) [readmission rate]. No study reported events regarding 30-day-mortality. In subgroup analyses and sensitivity analyses, TPB without PAP showed no significantly higher complication rates regarding all analyzed endpoints.

**Conclusions:**

Infectious complications after TPB occur very rarely and cannot be further reduced by PAP. Considering the results of this systematic review and adhering to the principles of effective antibiotic stewardship, omitting PAP in the context of TPB is advisable.

## Introduction

Prostate biopsy remains the primary and overwhelmingly dominant approach for diagnosing prostate cancer (PCa) and can be performed through both transrectal (TR) and transperineal (TP) approaches. Its histopathological findings play a crucial role in confirming PCa and are pivotal in planning subsequent therapy. Recent meta-analyses have emphasized the advantages of TP biopsy (TPB) for histologically confirming clinically significant PCa in the anterior region, without imposing limitations on other prostate zones [1, 2]. The seamless transition of Magnetic Resonance Imaging (MRI)/Transrectal Ultrasound (TRUS)-fusion prostate biopsy from TR to TP approach, conducted under local anesthesia, has demonstrated comparable oncological safety and no increase in post-interventional complications [3, 4]. Currently, guidelines from the American Urological Association (AUA) and the European Association of Urology (EAU) differ in their recommendations regarding the preferred approach for prostate biopsy [5–7]. While AUA guidelines consider both approaches as equivalent (conditional recommendation; evidence level: Grade C), EAU guidelines unequivocally favor TPB over TR biopsy (TRB) due to a lower risk of infectious complications (strong recommendation) [5–7]. It is widely acknowledged that TRB requires periprocedural antibiotic prophylaxis (PAP) [5–7]. Despite consistent adherence to PAP, ~5% infectious complications within a 30-day-timeframe are reported for TRB [8]. In the context of TPB, current recommendations of the EAU guidelines also still include PAP [6, 7]. However, data for this setting suggests that omitting PAP may not result in increased infectious complications [9, 10]. Two systematic reviews from 2021 and 2022 used different approaches to address the question whether PAP can be waived in the context of TPB [9, 10]. Basourakos et al. reviewed all 14 studies (up to December 2020) analyzing TPB without PAP (4772 patients) and compared infectious complications with 98 studies on TPB with PAP (37,805 patients) [9]. The rates of sepsis (0.05% vs. 0.08%; *p* = 0.2) and urinary tract infections/UTI (1.35% vs. 1.22%; *p* = 0.8) did not significantly differ between patients with or without PAP in the aggregated study cohorts [9]. In contrast, Castellani et al. exclusively analyzed studies enabling a direct comparison between TPB patients with and without PAP within each given study [10]. Until June 2021, they identified eight retrospective comparative studies, all providing a low certainty of evidence (2368 and 1294 patients with and without PAP, respectively), and derived pooled rates of sepsis (0.13% vs. 0.09%; *p* = 0.92) and UTI (0.11% vs. 0.31%; *p* = 0.29) [10].

Due to the limited evidence in current studies, neither AUA nor EAU guidelines currently recommend waiving PAP in the setting of TPB [5–7]. Subsequent to these reviews, additional studies, including randomized-controlled trials (RCT), have been published in the last three years, prompting us to re-evaluate current data to assess the need of PAP in the context of TPB.

Therefore, we conducted a systematic review including a meta-analysis, considering only studies with a direct comparison, to analyze and compare the pooled rates of infectious complications between patients with and without PAP in the context of TPB. In addition to pooling all available study data, we performed subgroup analyses considering the level of evidence (RCTs vs. others) and the antibiotics’ application scheme (single dose vs. no PAP and multiple doses vs. no PAP) based on data from available studies.

## Methods

### Approach to acquire evidence

We adhered to the 2020 Preferred Reporting Items for Systematic Reviews and Meta-Analyses (PRISMA) statement and utilized the Cochrane Handbook for systematic reviews of interventions [11, 12]. The study protocol was registered in PROSPERO (registration number: CRD42024500155) (Supplementary material).

### Literature search, study screening and selection

The clinical questions were structured using the PICOS (Population, Intervention, Comparator, Outcome, Study design) framework. The detailed PICOS design is available in Supplementary Table 1. Our inclusion criteria focused solely on studies comparing infection rates following TPB with vs. without PAP.

PubMed/Medline, Cochrane Central Controlled Register of Trials (CENTRAL), Embase, Web of Science, and a grey literature source (tripdatabase.com) were systematically explored up to 01 January 2024. The search utilized the following terms and Boolean operators: (‘transperineal’ OR ‘trans-perineal’) AND (‘prostate’ AND ‘biopsy’ AND ‘infection’). Details are listed in Supplementary Table 2. Studies were included based on predefined PICOS eligibility criteria. Case reports, editorials, letters to editors, and animal studies were excluded. No restrictions were applied regarding publication dates or language. Non-English and non-German articles were translated into German by professional translators.

Two investigators (M. Büchner, I.W.) performed an independent screening by title and abstract to identify ineligible reports. Potentially relevant reports underwent a full-text review, and their relevance was confirmed during the data extraction process. Any discrepancies were resolved by a third author (M.M.).

### Study endpoints and extracted variables

Combined rate of genitourinary tract infections, encompassing positive urine cultures with lower urinary tract symptoms, acute prostatitis, and/or epididymitis represented the primary outcome. Secondary outcomes comprised the aggregated rates of fever following TPB (defined as body temperature ≥38 °C), sepsis following TPB, readmission rates due to infections following TPB, and mortality following TPB resulting from infectious complications. Two investigators (M. Büchner, I.W.) independently extracted data from the included studies, focusing on the number of patients experiencing events across these endpoints, segmented by the administration or omission of PAP in the context of TPB. Any discrepancies were resolved by a third author (M.M.). Events across various endpoints were deemed relevant if occurring within 30 days following TPB.

The following patient and treatment characteristics (where available as mean or median) were recorded from the studies: first author’s name, publication year, country of study, study design, duration of the study period, patient demographics including age, PSA level, prostate volume, number of prostate cores sampled, number of biopsy-naive patients, number of patients diagnosed with prostate cancer, and details regarding the type and frequency of antibiotic administration (in the control arm, this corresponds to the patient group receiving PAP).

### Subgroup analysis

We pre-planned subgroup analyses for the primary study endpoint, considering the level of evidence of studies (RCTs or non-RCTs) and the frequency of antibiotic administration in the control arm (single dose vs. no-PAP and multiple doses vs. no-PAP). In response to growing antibiotic resistance concerns, a sensitivity analysis was conducted for the primary study endpoint, focusing exclusively on studies conducted after 2010.

### Statistical analysis

We summarized measures of patient and treatment characteristics from individual studies as median and interquartile range (IQR) across the included studies for the various variables under evaluation.

A meta-analysis was conducted to compare the incidence of the primary endpoint and four secondary endpoints among patients undergoing TPB without PAP (experimental arm) versus those undergoing TPB with PAP (control arm). The pooled incidences of these complications in each group were analyzed when two or more studies reported the same complication using identical parameters. Studies in which one of the specified endpoints was not investigated or for which no results were reported were not considered for this given endpoint. Studies in which the endpoint was examined but showed no events in both the experimental arm (no PAP) and the control arm (PAP) were considered for calculating the pooled incidence but were excluded from meta-analysis.

Differences in complication incidences were pooled using the Cochran–Mantel–Haenszel Method and expressed as odds ratio (OR) including 95%-confidence interval (CI). The OR was favored over the risk ratio (RR) as pre-calculation indicated low event rates for all endpoints, rendering the probability ratio more stable and informative compared to RR. In all odds ratio (OR) calculations, the non-PAP group was set as the reference category, with an OR > 1 indicating a higher probability of the endpoint in the PAP group, and an OR < 1 indicating a higher probability in the non-PAP group.

Irrespective of the calculated heterogeneity, random-effects models were computed due to the anticipated surplus of retrospective studies and the assumption that the true effect size could vary between studies. This approach allowed for better consideration of unmeasured sources of heterogeneity and variations between patients in the included study groups, thereby enhancing the robustness of the results. Standard error misfit was avoided, and the Restricted Maximum Likelihood (REML) was employed as an estimator for the variance of random effects. To assess study heterogeneity, we employed a chi-squared test with N-1 degrees of freedom, an *I*² test, and an alpha value of 0.1 for statistical significance. I² values of 25%, 50%, and 75% represented low, medium, and high degrees of heterogeneity, respectively.

Additionally, subgroup and sensitivity analyses were performed as mentioned above [13]. The homogeneity of the estimates within the subgroups and also within the sensitivity analysis groups was examined using the homogeneity measure Q, with a non-significant *p*-value indicating that the effects in studies from different groups do not substantially vary.

All reported p-values are two-sided, and statistical significance was defined as *p* < 0.05 for all tests. Data analysis was conducted using SPSS V.29 (IBM Corp., Armonk, NY, USA).

### Risk of bias and certainty of evidence assessment

Two authors (M. Büchner, I.W.) independently evaluated the quality and risk of bias (RoB) in the included studies. For RCTs, we utilized the RoB in randomized trials (ROB 2 tool), recommended by Cochrane Reviews and published in 2019 [14]. The overall RoB was categorized as ‘low,’ ‘some concerns,’ or ‘high.’ For non-randomized studies, the Newcastle-Ottawa Quality Assessment Scale for cohort studies was employed to assess RoB across three categories (selection, comparability, and outcome) [15]. As a result, RoB of each study was reported as a total score based on the aforementioned criteria. Any discrepancies were resolved through discussion or consultation with another author (M.M.).

To evaluate publication biases, we utilized funnel plots and Egger’s test (where *p* > 0.05 indicated no significant publication bias). The GRADE approach was employed to assess the certainty of evidence for each comparison, conducted by authors I.W. and M.M. [16, 17].

## Results

### Study selection and characteristics

**Fig. 1:**
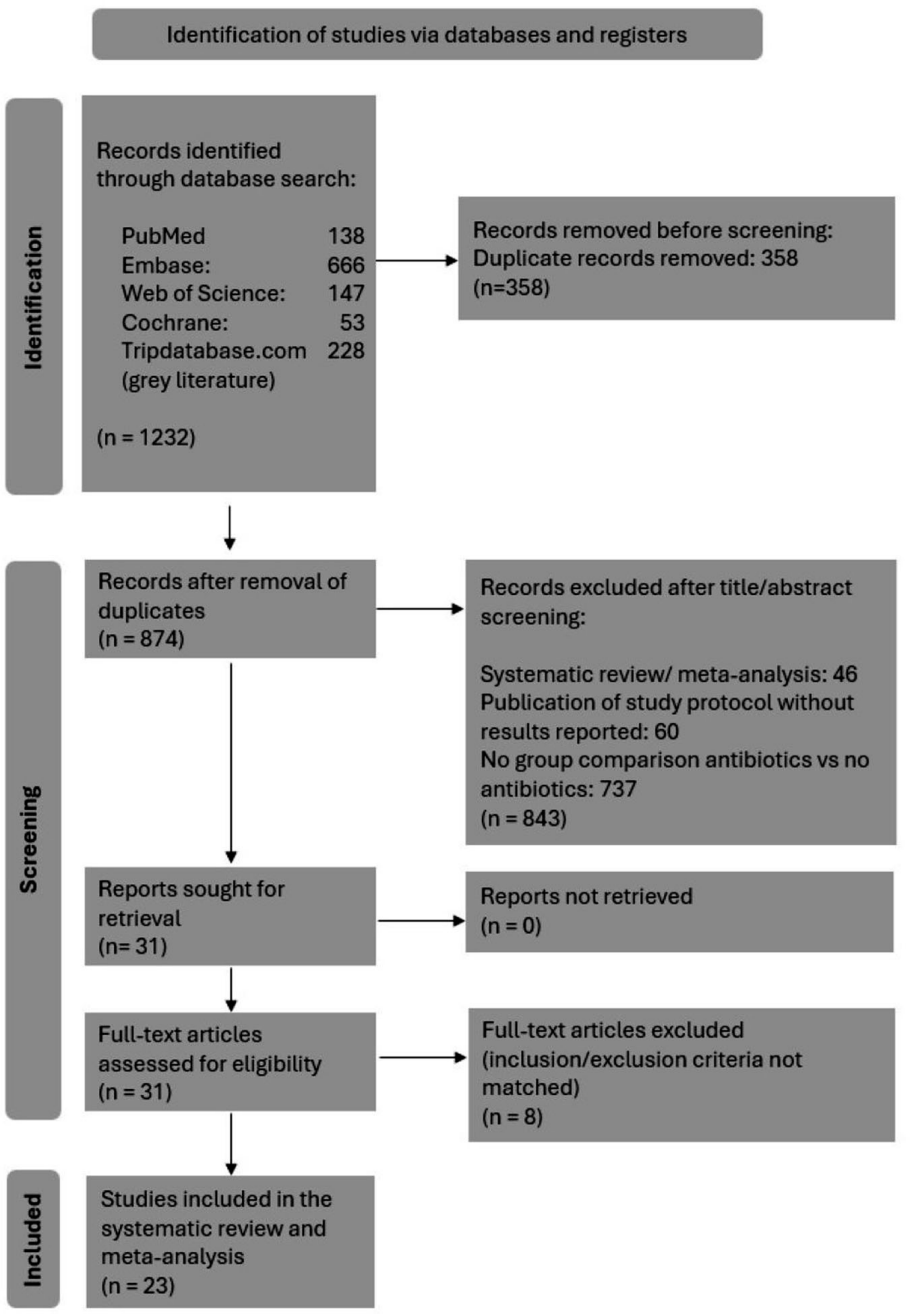
Preferred Reporting Items for Systematic Review and Meta-Analysis (PRISMA) 2020 flow diagram showing the study selection process with inclusion and exclusion criteria for the studies reviewed [11].

Ultimately, 23 studies with 12,324 patients were included in the systematic review. Studies were categorized into RCTs (*n* = 2), prospective studies (*n* = 9), and retrospective cohort studies (*n* = 12). Among these, 6520 patients received PAP as part of the treatment protocol, while 5804 patients (47.1% of the total group) did not. Study selection process is illustrated in Fig. 1, and reasons for exclusion of trials upon full-text evaluation are listed in Supplementary Table 3. Characteristics of included studies are detailed in Table 1. Twelve studies were conducted in the United States, 10 in European countries, and one in China. 21 of 23 studies (91.3%) were conducted after 2010. Whether single or multiple administrations of PAP were administered was reported in only 16 of the 23 studies, with 12 of these 16 studies (75%) involving a single dose administration.

**Table 1:** Characteristics of studies included according to the inclusion / exclusion criteria.

First author	Year of publication	Number of patients without PAP	Number of patients with PAP	Country	Duration of study (month)	Study type	Type of pain control	Dosage of PAP in the control group	Route of PAP administration in the control group
RCT									
Chernyscheva [34]	2021	35	50	RUS	12	RCT	LA	SIN	IV/IM
Jacewicz [35]	2022	276	277	INT	15	RCT	LA	SIN	VAR
NRS									
Packer [36]	1984	44	118	USA	48	R	NR	MUL	VAR
Lee [37]	1986	20	25	USA	7	R	LA	NR	NR
Ristau [38]	2018	400	600	USA	81	R	DIF	SIN	OR
Wetterauer [39]	2020	177	223	SUI	53	R	LA	MUL	OR
John [40]	2021	294	149	GBR	16	P	LA	SIN	VAR
Jacewicz [41]	2021	229	148	INT	19	R	LA	MUL	VAR
Lopez [42]	2021	175	1043	INT	24	P	LA	SIN	VAR
Szabo [43]	2021	212	92	USA	79	R	LA	SIN	VAR
Bianco [44]	2021	480	728	USA	48	P	NR	SIN	VAR
Briggs [45]	2021	68	62	USA	16	R	LA	NR	VAR
Cricco-Lizza [46]	2021	45	81	USA	29	P	DIF	MUL	COM
Setia [47]	2021	450	538	USA	98	R	DIF	SIN	OR
Wertheimer [48]	2021	49	25	USA	NR	R	LA	NR	NR
De Vulder [49]	2022	103	100	BEL	15	P	LA	SIN	NR
Ginsburg [50]	2022	282	123	USA	74	R	LA	NR	NR
He [51]	2022	291	249	CHN	41	R	GA	SIN	IV/IM
Pedersen [52]	2022	133	10	DEN	8	P	LA	SIN	OR
Walter [53]	2022	152	76	SUI	30	P	GA	NR	NR
Akinsola [54]	2023	217	235	USA	NR	P	NR	NR	NR
Dhir [55]	2023	669	1543	USA	NR	R	NR	NR	NR
Honoré [56]	2023	1003	25	NOR	48	P	DIF	SIN	OR

*BEL* Belgium, *CHN* China, *COM* combined (orally/intravenously/intramuscularly), *DEN* Denmark, *DIF* different anesthetic procedures, *GA* general anesthesia, *GBR* Great Britain, *INT* international, *IV/IM* intravenously or intramuscularly (but no combination), *LA* local anesthesia, *MUL* multiple doses, *NOR* Norway, *NR* not reported, *NRS* non-randomized studies, *OR* oral, *P* prospective, *PAP* periprocedural antibiotic prophylaxis, *R* retrospective, *RCT* randomized controlled trials, *RUS* Russia, *SIN* single dose, *SUI* Switzerland, *USA* United States of America, *VAR* various routes of PAP administration.

### Pooled comparison and meta-analysis of genitourinary tract infections following TPB

The endpoint ‘genitourinary tract infections following TPB’ was investigated by 21 of 23 studies. An event rate of 0.37% (20/5417) was observed among patients without PAP and of 0.50% (27/5385) among patients receiving PAP (*p* = 0.297).

**Fig. 2:**
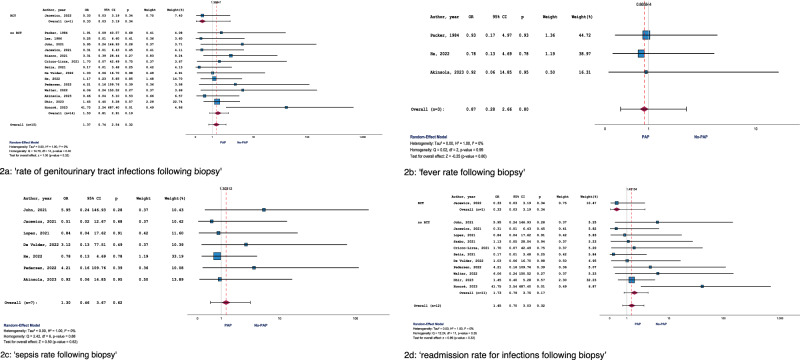
Forest plots of the meta-analyses for the proportion of patients who underwent transperineal prostate biopsy with vs. without receiving periprocedural prophylactic antibiotics. The following endpoints were reported: **a** ‘rate of genitourinary tract infections following biopsy’, **b** ‘fever rate following biopsy’, **c** ‘sepsis rate following biopsy’, **d** ‘readmission rate for infections following biopsy’.

Fifteen studies, including one RCT, were eligible for meta-analysis. Pooled OR across these 15 studies was 1.37 (95% CI: 0.74–2.54; *p* = 0.319) with *I*² = 0% (Fig. 2a). The RCT providing evidence for this endpoint revealed an OR of 0.33 (95% CI: 0.03–3.19; *p* = 0.340), while meta-analysis of all available NRS showed an aggregated OR of 1.53 (95% CI:0.81–2.91; *p* = 0.190) (Fig. 2a). In terms of sensitivity analysis, two studies were conducted before 2010 (OR: 0.74, 95% CI:0.08–6.90; *p* = 0.794), while 13 studies were performed after 2010 (OR: 1.44, 95% CI: 0.76–2.74; *p* = 0.266). A high level of homogeneity was observed between the pooled results of studies from both time periods (*Q* = 0.312; *p* = 0.576).

### Pooled comparison and meta-analysis of fever following TPB

The endpoint ‘fever as a consequence of TPB’ was investigated by 14 of 23 studies. An event rate of 0.26% (9/3496) was calculated for patients without PAP and of 0.44% (10/2,251) for patients receiving PAP (*p* = 0.228).

Only three studies (no RCT) were eligible for meta-analysis. OR across these three studies was 0.87 (95% CI: 0.28–2.66; *p* = 0.801) with *I*² = 0% (Fig. 2b). In terms of sensitivity analysis, one study was conducted before 2010 (OR: 0.93, 95% CI: 0.17–4.97; *p* = 0.932), while two studies were performed after 2010 (OR: 0.82, 95% CI: 0.18–3.70; *p* = 0.794). A high level of homogeneity existed between the pooled results of studies from both time periods (*Q* = 0.012; *p* = 0.912).

### Pooled comparison and meta-analysis of sepsis following TPB

The endpoint ‘sepsis following TPB’ was investigated by 21 of 23 studies. An event rate of 0.13% (6/4655) was found among patients without PAP and of 0.16% (7/4249) among patients with PAP (*p* = 0.658).

Seven studies were eligible for meta-analysis. Pooled OR across these 7 studies was 1.30 (95% CI: 0.46–3.67; *p* = 0.616) with *I*² = 0% (Fig. 2c). All studies related to this endpoint were performed after 2010, rendering sensitivity analyses unnecessary in this context.

### Pooled comparison and meta-analysis of readmission rate for infections following TPB

The endpoint ‘readmission rate for infections following TPB’ was investigated by 19 of 23 studies. An event rate of 0.29% (14/4772) was detected among patients without PAP and of 0.35% (18/5160) among patients with PAP (*p* = 0.626).

Twelve studies, including one RCT, were eligible for meta-analysis. OR across these 12 studies was 1.45 (95% CI: 0.70–3.03; *p* = 0.320) with *I*² = 0% (Fig. 2d). The RCT providing evidence for this endpoint revealed an OR of 0.33 (95% CI: 0.03–3.19; *p* = 0.340), while meta-analysis of all available NRS showed an aggregated OR of 1.73 (95% CI: 0.79–3.75; *p* = 0.170) (Fig. 2d). All studies related to this endpoint were conducted after 2010, rendering sensitivity analyses unnecessary in this context.

### Pooled comparison and meta-analysis of death (mortality) due to an infectious complication of TPB

The endpoint ‘death due to an infectious complication of TPB’ was reported by all 23 studies, with no events observed in either patient group (with vs. without PAP). Therefore, all statistical analyses for this endpoint were considered unnecessary.

### Subgroup analysis by level of evidence of studies (RCT versus no RCT) and application scheme of the antibiotics (single versus multiple dose administration of PAP)

For each of the four endpoints eligible for meta-analyses, two subgroup analyses were conducted: one based on the level of evidence of studies and the other based on the frequency of antibiotic administration in the control arm.

For the first endpoint (‘genitourinary tract infections’), one RCT and 14 non-RCT studies were included in the meta-analysis. The study results of the RCT (OR: 0.33, 95% CI: 0.03–3.19; *p* = 0.338) and the pooled results of the non-RCTs (OR: 1.53, 95% CI: 0.81–2.91; *p* = 0.192) exhibited a sufficient level of homogeneity (*Q* = 1.631; *p* = 0.202). For this endpoint, 11 studies on the frequency of antibiotic administration in the control arm were included in the subgroup analysis, comprising 8 studies with PAP single dose administration and 3 studies with multiple dose administration of PAP. Pooled results for single dose administration of PAP (OR: 1.78, 95% CI: 0.60–5.31; *p* = 0.300) and for multiple dose administration of PAP (OR: 0.98, 95% CI: 0.16–5.86; *p* = 0.979) demonstrated sufficient homogeneity between them (*Q* = 0.316; *p* = 0.574).

For the second endpoint (‘fever’), no RCT was available, rendering a subgroup analysis unnecessary. For this endpoint, one study with single dose administration of PAP (OR: 0.78, 95% CI: 0.13–4.69; *p* = 0.784) and one study with multiple dose administration of PAP (OR: 0.93, 95% CI: 0.17–4.97; *p* = 0.932) were analyzable. Homogeneity of the estimates in these two studies was observed (*Q* = 0.020; *p* = 0.887).

For the third endpoint (‘sepsis’), no RCT was available, rendering a corresponding subgroup analysis unnecessary. For this endpoint, five studies with single-dose administration of PAP (OR: 1.58, 95% CI: 0.48–5.19; *p* = 0.452) and one study with multiple dose administration of PAP (OR: 0.51, 95% CI: 0.02–12.67; *p* = 0.683) were analyzable. Homogeneity of the estimates in these two studies was observed (*Q* = 0.415; *p* = 0.520).

For the fourth endpoint (‘readmission rate for infections following biopsy’), one RCT and 11 non-RCT studies were included in the meta-analysis. The study results of the RCT (OR: 0.33, 95% CI: 0.03–3.19; *p* = 0.338) and pooled results of the non-RCTs (OR: 1.73, 95% CI: 0.79–3.75; *p* = 0.168) exhibited a sufficient level of homogeneity (*Q* = 1.830; *p* = 0.176). For this endpoint, 10 studies on the frequency of antibiotic administration in the control arm were included in the subgroup analysis, comprising 8 studies with single-dose administration of PAP and 2 studies with multiple dose administration of PAP. Pooled results for single-dose administration of PAP (OR: 1.55, 95% CI: 0.43–5.58; *p* = 0.499) and for multiple dose administration of PAP (OR: 0.69, 95% CI: 0.07–6.27; *p* = 0.739) demonstrated sufficient homogeneity between them (*Q* = 0.393; *p* = 0.531).

### Risk of bias and certainty of evidence assessment

There were no indications of substantial publication bias for any of the endpoints based on the Funnel Plots (Supplementary Fig. 1a–d) or according to the Egger tests (*p*-values ranged between 0.517 and 0.950). Results of the RoB assessment are shown in Supplementary Table 4 (for RCTs) and Supplementary Table 5 (for non-RCTs). Certainty of evidence for all endpoints is displayed in Table 2 and Supplementary Table 6.

**Table 2:** GRADE Summary of findings.

Endpoint	Studies and participants	Relative effect OR (95%-CI)	Anticipated absolute effect (95%-CI)		Difference (95%-CI)	Certainty^a^	What happens (standardized GRADE terminology)
			CG (with PAP)	IG (without PAP)			
GUI	1 RCT (*n* = 553) 14 NRS (*n* = 8155)	0.330 (0.034–3.189) 1.533 (0.807–2.914)	0.361 (–0.346–1.068) 0.646 (0.399–0.893)	1.087 (–0.139–2.313) 0.412 (0.216–0.606)	0.726 (-0.683–2.135) –0.234 (–0.572–0.103)	Low	There is no evidence that omitting PAP in the context of TPB is associated with a higher rate of GUI.
Fever	3 NRS (*n* = 1154)	0.866 (0.282–2.659)	1.329 (0.420–2.238)	1.087 (0.225–1.949)	–0.242 (–1.497–1.013)	Very low	There is no evidence that omitting PAP in the context of TPB is associated with a higher rate of fever.
Sepsis	7 NRS (*n* = 3376)	1.303 (0.463–3.670)	0.362 (0.095–0.629)	0.416 (0.084–0.749)	0.054 (–0.377–0.486)	Very low	There is no evidence that omitting PAP in the context of TPB is associated with a higher rate of sepsis.
Readmission	1 RCT (*n* = 553) 11 NRS (*n* = 7270)	0.330 (0.034–3.189) 1.726 (0.794–3.751)	0.361 (–0.346–1.068) 0.447 (0.235–0.659)	1.087 (–0.139–2.313) 0.317 (0.130–0.504)	0.726 (–0.683–2.135) –0.130 (–0.414–0.155)	Low	There is no evidence that omitting PAP in the context of TPB is associated with a higher rate of readmission.

*CG* comparison group, *CI* confidence interval, *GUI* genitourinary tract infections, *GRADE* grading of recommendations assessment, development, and evaluation, *IG* intervention group, *NRS* non-randomized studies, *OR* odds ratio, *PAP* periprocedural antibiotic prophylaxis, *RCT* randomized controlled trials, *TPB* trans-perineal biopsy.

*Note:* For GRADE, only those studies were considered that were included in the meta-analyses and had events in the endpoints.

^a^GRADE category of evidence (an evaluation was conducted here that considered the results of both RCT and NRS):

*High certainty* (we are very confident that the true effect lies close to that of the estimate of the effect).

*Moderate certainty* (we are moderately confident in the effect estimate; the true effect is probably close to the estimate, but it is possibly substantially different).

*Low certainty* (our confidence in the effect estimate is limited; the true effect could be substantially different from the estimate of the effect).

*Very low certainty* (we have very little confidence in the effect estimate; the true effect is likely to be substantially different from the estimate of effect).

## Discussion

The current systematic review and meta-analysis represents the first study world-wide which directly compares infectious complications in patients who underwent TPB with and without PAP based on both randomized and non-randomized trials. Our aggregated analysis of the current data represents an update to the methodological approach of Castellani et al. (2021) [10]. Whereas their study included eight non-randomized trials, we have now expanded our systematic review to encompass 21 non-randomized studies and two randomized controlled trials (RCTs). No statistically significant differences concerning all analyzed infectious endpoints (genitourinary infections, fever, sepsis, and readmission rates due to infectious complications) were observed following TPB with and without PAP. Our findings not only corroborate the results of the systematic review by Castellani et al. but also align with the insights from a methodologically distinct systematic review by Basourakos et al. (for a comparison of the methodology and results of the three systematic reviews now available on this topic, Table 3) [9, 10]. However, our study advances the discussion by incorporating newer studies including RCT and offering a more robust statistical analysis, thereby enhancing the reliability of the conclusions.

**Table 3:** Presentation of the pooled incidence rates of infectious complications following transperineal prostate biopsy in groups with versus without periprocedural antibiotic prophylaxis (PAP) derived from all systematic reviews currently available.

Criteria	Basourakos [9]	Castellani [10]	Our own systematic review
Methodology of the systematic review	Indirect comparison of study results	Direct comparison of study results	Direct comparison of study results
Completion date of the literature search	29-Dec-2020	02-Jun-2021	01-Jan-2024
Number of included studies	106	8	23
Number of included studies with a prospective-randomized design^a^	Not applicable	0	2
Number of patients with PAP	37,805	2368	6520
Number of patients without PAP	4772	1294	5804
Genitourinary tract infections (with vs. without PAP)	1.35% (403/29,880) vs. 1.22% (58/4772), *p* = 0.494^b^	0.13% (3/2368) vs. 0.31% (4/1294), *p* = 0.252^b^	0.37% (20/5417) vs. 0.50% (27/5385), *p* = 0.297
Fever (with vs. without PAP)	Not reported	0.69% (5/725) vs. 0.47% (4/846), *p* = 0.740^b^	0.26% (9/3496) vs. 0.44% (10/2251), *p* = 0.228
Sepsis (with vs. without PAP)	0.05% (19/37,805) vs. 0.08% (4/4772), *p* = 0.318^b^	0.13% (3/2368) vs. 0.08% (1/1294), *p* = 0.999^b^	0.13% (6/4655) vs. 0.16% (7/4249), *p* = 0.658
Readmission rate (with vs. without PAP)	Not reported	0.13% (3/2368) vs. 0.23% (3/1294), *p* = 0.433^b^	0.29% (14/4772) vs. 0.35% (18/5160), *p* = 0.626
30-day mortality (with vs. without PAP)	Not reported	Each 0%	Each 0%

*PAP* periprocedural antibiotic prophylaxis.

^a^Randomized studies regarding the trial question ‘with versus without PAP’.

^b^The *p*-values were independently calculated using Fisher’s exact test (Chi-square) as they were not reported in the original study.

It is indisputable that our medical practices and the administration of antibiotics significantly influence the development of bacterial resistance. Therefore, every instance of forgoing PAP can be regarded as a success, potentially counteracting recent negative trends. In 2022, the ‘Antimicrobial Resistance Collaborators’ published their GRAM Report in ‘Lancet’, estimating that nearly 5 million deaths worldwide in 2019 were closely associated with antimicrobial resistance (AMR), with ~1.3 million deaths directly attributable to AMR. Their analyses identified *Escherichia coli* as the most significant bacterium contributing to these substantial mortality rates [18].

These developments are particularly noteworthy in the context of prostate biopsy, as the global incidence of prostate cancer (and consequently the frequency of histological confirmations through biopsy) is projected to double by 2040 [19]. Thus, from pragmatic and forward-looking perspectives, the transperineal biopsy approach, which current studies suggest does not require PAP, should be favored over the transrectal biopsy approach, where PAP will always be necessary [20]. Nonetheless, several high-confidence studies currently demonstrate that this ‘apples-to-oranges comparison’ shows no inferiority in infectious endpoints for PAP-free transperineal biopsy compared to PAP-accompanied transrectal biopsy. For instance, Hu et al. published a randomized-controlled study comparing infectious complications following TPB without PAP vs. TRB with PAP [21]. Based on an entire cohort of 658 patients, they found no higher infection rates following TPB opposed to TRP. Of note, in this study, a rectal culture to screen for fluoroquinolone-resistant organisms was obtained prior to transrectal biopsy in all patients and targeted PAP was administered. It seems questionable whether such an effort is feasible in all patients undergoing TRP in daily routine. Furthermore, the detection rate of clinically significant cancer was similar in both groups indicating comparable diagnostic accuracy of both approaches. Although no statistically significant differences in infection rates were initially observed due to an insufficient number of enrolled patients, the potential clinical impact of even a 1% reduction in infection rates associated with prostate biopsy seems noteworthy, given the millions of procedures performed annually worldwide [22]. Interestingly, final results of this study published after meeting the sample size requirement even yielded a statistically significant difference in infection rates in favor of TPB [23]. In another RCT including 718 patients, Mian et al. reported no statistically significant differences in infectious or non-infectious complications following TPB without PAP as opposed to TRB including augmented PAP [24].

Based on their longitudinal cohort study, Newman et al. concluded that targeted antimicrobials based on rectal swab culture failed to reduce the overall risk of post TRB sepsis as opposed to TRB including empirical PAP, while a change towards TPB nearly eliminated this risk [25]. The RCT conducted by Ploussard et al. yielded comparable detection rates and complications associated with TRP and TPB in a total of 270 patients, respectively [26]. In contrast, Diamand et al. found an even better detection rate concerning aggressive tumors for TPB compared to TRB in their trial comprising a total cohort of 3949 patients [27]. These studies show comparable oncologic results and non-infectious complications of TRB and TPB, respectively. In terms of infectious complications, omitting PAP in the context of TPB does not result in higher complications as opposed to prostate biopsy with PAP (regardless the PAP approach used).

Moreover, abandonment of PAP in the context of TPB offers further advantages. Recent studies underline the impact of the microbiome on patients’ outcome, e.g., by modifying the efficacy of systemic treatments in various tumors [28–30]. This suggests that a potential deterioration of patients’ microbiome following PAP may adversely affect further prognosis—a risk that can be excluded by omitting PAP.

In summary, our study yields substantial results suggesting that emerging recommendations to abstain from PAP in patients undergoing TPB are warranted. By reducing unnecessary antibiotic administration, healthcare providers can mitigate the risks associated with antibiotic resistance, which represents a growing global health concern. Furthermore, this approach aligns with the principles of antibiotic stewardship and promotes more sustainable healthcare practices without compromising patient safety.

Despite the strengths of our analysis, several limitations must be considered. The primary limitation is the lack of more high-quality studies and the inclusion of studies with mixed methodological designs. This could introduce bias, as data from RCTs and prospective studies are likely to have been collected more intensively and reliably than data from retrospective studies. To address this, we analyzed RCTs and NRS separately. Additionally, the overall rate of infectious complications was low across all studies, regardless of methodological design, suggesting that this factor is unlikely to have skewed our aggregated analyses. However, not all studies investigated each of the five selected endpoints, limiting our ability to draw definitive conclusions about specific infectious complications. Moreover, the absence of long-term follow-up data constrains our understanding of delayed complications, which are crucial for comprehensive guideline recommendations.

Another limitation is that our analyses did not account for potential patient-level differences, such as medical comorbidities, prior exposure to prostate biopsy, or the number of previous prostate biopsies—factors that could contribute to post-procedural infections.

An additional limitation, which might also enhance the generalizability of our results, is the variation in PAP regimens and doses in the comparator arms. The antibiotics used, as well as their routes and durations of administration, varied significantly between studies. Consequently, we chose to conduct all meta-analyses as random-effect models despite a uniform *I*² of 0%. It is also important to note that the management of readmission (potentially as hospitalization) due to infectious complications after prostate biopsy varies greatly worldwide and is thus difficult to standardize. Finally, we were unable to assess the impact of the number of biopsy cores taken on the post-procedural infection rate, as several studies did not report this parameter.

These extensive limitations, considered alongside our risk-of-bias assessment, ultimately led us to rate the certainty of our analyses for each endpoint as ‘low’ or ‘very low’ according to the GRADE approach. We could not analyze the endpoint ‘Mortality’ at all, as none of the studies reported relevant events.

Future research should focus on addressing the gaps identified in this review. Additional RCTs with larger sample sizes and standardized antibiotic administration protocols are desirable to further enhance the level of evidence thereby increasing the certainty of conclusions. Moreover, investigating the role of patient-specific factors, such as comorbidity profiles and previous antibiotic usage, could help tailor ABS strategies more effectively. Longitudinal studies are also essential to assess the long-term safety and efficacy of omitting PAP. Some of the features listed above are already addressed by two ongoing studies registered at ClinicalTrials.gov with no results available yet: a prospective cohort study from Hong Kong (NCT06359964) and an RCT from Indonesia (NCT04985110).

Alternative imaging techniques for initial diagnosis of PCa are currently investigated to replace or complement MRI in this setting [31]. Preliminary evidence supports initiation of curative treatment for PCa based on MRI in combination with Prostate-Specific Membrane Antigen Ligand Positron Emission Tomography/Computed Tomography (PSMA-PET-CT) without prior biopsy in selected patients [32, 33]. In the future, this approach may ultimately prevent any infectious complications following diagnostic work-up for PCa.

## Conclusions

The systematic review, encompassing over 12,000 men, included 23 studies comparing TPB with versus without PAP in terms of post-interventional infectious complications. No significant group differences were identified in the aggregated analysis or various subgroup analyses for these endpoints. As only two of these studies were RCTs, resulting in a substantial RoB for many studies, the recommendation to omit PAP during TPB can only be made with (very) low certainty. Conversely, the consistency of results across studies suggests that, in the context of progressive Antibiotic Stewardship, the omission of PAP in this setting poses a negligible risk to patients. The results of an additional RCT are anticipated, which will facilitate a reassessment.

## Supplementary information

Supplementary data
