# Supplementary material for: Infectious complications following transperineal prostate biopsy with or without periprocedural antibiotic prophylaxis—a systematic review including meta-analysis of all comparative studies
Source: Prostate Cancer Prostatic Dis. 2024 Dec 31;29(1):47–56. doi: 10.1038/s41391-024-00934-9 (PMC12909119; doi:10.1038/s41391-024-00934-9)
Supplement: Supplementary file 1 — Supplementary data [file 41391_2024_934_MOESM1_ESM.docx]

**Prostate Cancer and Prostatic Diseases**

**Supplementary data**

**Supplement to Wolff I, Büchner M, Hauner K, et al.**

**Infectious complications following transperineal prostate biopsy with or without periprocedural antibiotic prophylaxis – a systematic review including meta-analysis of all comparative studies**

**Table of content**

[1. Authors 3](#_Toc180082794)

[2. PROSPERO Registration 4](#_Toc180082795)

[3. Clinical questions using the PICOS (Population, Intervention, Comparator, Outcome, Study design) framework (Supplementary Table 1) 5](#_Toc180082796)

[4. Search strategies (Supplementary Table 2) 6](#_Toc180082797)

[5. Studies screened, but excluded from the comprehensive review after full-text evaluation (Supplementary Table 3) 8](#_Toc180082798)

[6. Risk of bias assessment of randomized controlled trials using the RoB2-tool
(Supplementary Table 4) 9](#_Toc180082799)

[7. Risk of bias assessment of non-randomized studies using the Newcastle-Ottawa scale (Supplementary Table 5) 10](#_Toc180082800)

[8. Evidence Profiles (Supplementary Table 6) 11](#_Toc180082801)

[9. Funnel plots including Egger’s tests for the different endpoints (Supplementary Figures 1a-d) 14](#_Toc180082802)

[10. PRISMA 2020 for Abstracts Checklist (Supplementary Table 7) 16](#_Toc180082803)

[11. PRISMA 2020 checklist (Supplementary Table 8) 17](#_Toc180082804)

[12. References 22](#_Toc180082805)

# 1. Authors

| Author | Degree | Affiliation | Telephone number | Email |
| --- | --- | --- | --- | --- |
| Ingmar Wolff | MD | Department of Urology, University Medicine Greifswald, Greifswald, Germany | +49 3834  86 5979 | Ingmar.Wolff@  med.uni-greifswald.de |
| Markus Büchner | Mr. | Department of Urology, University Medicine Greifswald, Greifswald, Germany | +49 3834  86 5979 | Markus.Buechner@  med.uni-greifswald.de |
| Katharina Hauner | MD | Department of Urology, School of Medicine and Health, TUM University Hospital, Technical University of Munich, Munich, Germany | +49 89  4140 2521 | Katharina.Hauner@  mri.tum.de |
| Florian Wagenlehner | MD, Prof. | Department of Urology, Pediatric Urology and Andrology, Justus Liebig University Giessen, Giessen, Germany | +49 641  985 44501 | Florian. Wagenlehner@  chiru.med.uni-giessen.de |
| Martin Burchardt | MD, Prof. | Department of Urology, University Medicine Greifswald, Greifswald, Germany | +49 3834  86 5979 | Martin.Burchardt@  med.uni-greifswald.de |
| Marianne Abele-Horn | MD, Prof. | Institute for Hygiene and Microbiology, University of Würzburg, Würzburg, Germany | +49 931  31 46161 | Marianne.  Abele-Horn@uni-wuerzburg.de |
| Bernd Wullich | MD, Prof. | Department of Urology and Pediatric Urology, University Hospital Erlangen, Friedrich-Alexander Universität Erlangen-Nürnberg, Erlangen, Germany  Comprehensive Cancer Center Erlangen-EMN (CCC ER-EMN), Erlangen, Germany | +49 9131  85 33683 | bernd.wullich@ uk-erlangen.de |
| Christian Gilfrich | MD, Assoc.-Prof. | Department of Urology, St. Elisabeth Hospital Straubing, Brothers of Mercy Hospital, Straubing, Germany | +49 9421  710 1701 | Christian.Gilfrich@  klinikum-straubing.de |
| Adrian Pilatz | MD, Prof. | Department of Urology, Pediatric Urology and Andrology, Justus Liebig University Giessen, Giessen, Germany | +49 641  985 44501 | Adrian.Pilatz@  chiru.med.uni-giessen.de |
| Matthias May | MD, Prof. | Department of Urology, St. Elisabeth Hospital Straubing, Brothers of Mercy Hospital, Straubing, Germany | +49 9421  710 1701 | Matthias.May@  klinikum-straubing.de |

# 2. PROSPERO Registration

This study was registered in the Prospero database (CRD42024500155) on 18-Jan-2024.

# 3. Clinical questions using the PICOS (Population, Intervention, Comparator, Outcome, Study design) framework (Supplementary Table 1)

| Element | Focus |
| --- | --- |
| Clinical question | Can PAP be omitted during TPB without a significantly higher rate of infectious complications? |
| Population | Adult men undergoing a TPB for the histological exclusion or detection of prostate cancer |
| Intervention | Omission of PAP in the context of TPB |
| Comparator | Administration of PAP during TPB |
| Outcomes | Rate of genitourinary tract infections following TPB, fever as a consequence of TPB, sepsis following TPB, readmission rate for infections following TPB, and mortality due to an infectious complication of TPB within a period of 30 days following TPB |
| Study design | prospective and retrospective comparative observational studies and randomized controlled trials |
| Databases searched | PubMed/Medline, Cochrane Central Controlled Register of Trials (CENTRAL), Embase, Web of Science, and a grey literature source (tripdatabase.com) |
| Timeframe | No time limit |
| Geography | No geographic limit |
| Language | No restriction on publication languages. Non-English and non-German articles were translated into German by professional translators. |
| Eligibility criteria | All identified full-text articles and conference abstracts, regardless of the language of publication, were included if they compared infectious complications in men undergoing TPB in groups without PAP versus with PAP within a single study |
| Exclusion criteria | Case reports, editorials, letters to editors, animal and pediatric studies, as well as all studies without a direct comparison between the aforementioned intervention and the comparator, were excluded |
| Risk of bias assessment | Cochrane Collaboration’s RoB 2.0 tool was used for randomized trials and Newcastle-Ottawa-Scale tool for non-randomized observational studies |

**Legend:** **PAP**, periprocedural antibiotic prophylaxis; **TPB**, transperineal prostate biopsy

# 4. Search strategies (Supplementary Table 2)

The following terms were used for the search of studies meeting the predefined inclusion criteria: (“transperineal” OR “trans-perineal”) AND (“prostate” AND “biopsy” AND “infection”). Databases were systematically explored up to 01-January-2024.

Search strategy for PubMed/Medline

| Search term | **Hits** |
| --- | --- |
| #1: (“transperineal” OR “trans-perineal”) | 3,539 |
| #2: (“prostate”) | 238,580 |
| #3: (“biopsy”) | 508,860 |
| #4: (“infection”) | 1,564,946 |
| #5: #2 AND #3 AND #4 | 845 |
| #6: #1 AND #5 | 138 |

Search strategy for Cochrane Central Controlled Register of Trials (CENTRAL)

| Search term | **Hits** |
| --- | --- |
| #1: (“transperineal” OR “trans-perineal”) | 410 |
| #2: (“prostate”) | 25,175 |
| #3: (“biopsy”) | 36,847 |
| #4: (“infection”) | 151,566 |
| #5: #2 AND #3 AND #4 | 306 |
| #6: #1 AND #5 | 53 |

Search strategy for Embase

| Search term | **Hits** |
| --- | --- |
| #1: (“transperineal” OR “trans-perineal”) | 6,669 |
| #2: (“prostate”) | 428,343 |
| #3: (“biopsy”) | 1,083,501 |
| #4: (“infection”) | 3,060,737 |
| #5: #2 AND #3 AND #4 | 3,326 |
| #6: #1 AND #5 | 666 |

Search strategy for Web of Science

| Search term | **Hits** |
| --- | --- |
| #1: (“transperineal” OR “trans-perineal”) | 4,440 |
| #2: (“prostate”) | 339,045 |
| #3: (“biopsy”) | 352,874 |
| #4: (“infection”) | 1,755,505 |
| #5: #2 AND #3 AND #4 | 848 |
| #6: #1 AND #5 | 147 |

Search strategy for tripdatabase.com (grey literature)

| Search term | **Hits** |
| --- | --- |
| #1: (“transperineal” OR “trans-perineal”) | 1,484 |
| #2: (“prostate”) | 74,018 |
| #3: (“biopsy”) | 132,968 |
| #4: (“infection”) | 567,701 |
| #5: #2 AND #3 AND #4 | 3,596 |
| #6: #1 AND #5 | 228 |

# 5. Studies screened, but excluded from the comprehensive review after full-text evaluation (Supplementary Table 3)

**Legend:** **PAP**, periprocedural antibiotic prophylaxis

| First author and year of study | Reason for exclusion |
| --- | --- |
| Boeve 2023 [1] | Endpoints for subgroups (PAP vs. no PAP) not reported |
| Mian 2023 [2] | Transperineal biopsy was compared to transrectal biopsy. Therefore, inclusion criteria of this systematic review/ meta-analysis were not met. |
| Hughes 2022 [3] | Endpoints not reported |
| Setia 2022 [4] | Number of patients receiving antibiotics not reported |
| Chernysheva 2022 [5] | Inadequate randomization (additional information were provided by the authors) |
| Alidjanov 2021 [6] | Number of patients receiving antibiotics not reported |
| Günzel 2021 [7] | Endpoints for patients receiving antibiotics not reported |
| John 2022 [8] | Represents a cohort included in the study of John et al. (2021), which is incorporated in this systematic review / meta-analysis |

# 6. Risk of bias assessment of randomized controlled trials using the RoB2-tool (Supplementary Table 4)

|  | **First author and year of study** | **D1** | **D2** | **D3** | **D4** | **D5** | **Overall** |
| --- | --- | --- | --- | --- | --- | --- | --- |
| 1 | Chernysheva 2021 [9] |  |  |  |  |  |  |
| 2 | Jacewicz 2022 [10] |  |  |  |  |  |  |
|  | | Low risk | | | | | |
|  | | Some concerns | | | | | |
|  | | High risk | | | | | |
| D1 | | Randomization process | | | | | |
| D2 | | Deviations from the intended interventions | | | | | |
| D3 | | Missing outcome data | | | | | |
| D4 | | Measurement of the outcome | | | | | |
| D5 | | Selection of the reported result | | | | | |

**Legend:** **D**, domain

# 7. Risk of bias assessment of non-randomized studies using the Newcastle-Ottawa scale (Supplementary Table 5)

| First Author | Year | Selection | | | | Comparability | Outcome | | | Total score |
| --- | --- | --- | --- | --- | --- | --- | --- | --- | --- | --- |
|  |  | **Represen-tativeness of the exposed cohort** | **Selection of the non exposed cohort** | **Ascertain-ment of exposure** | **Demonstration that outcome of interest was not present at start of study** | **Comparability of cohorts on the basis of the design or analysis** | **Assess-ment of outcome** | **Was follow-up long enough for outcomes to occur** | **Adequacy of follow- up of cohorts** |  |
| Packer [11] | 1984 | ⋆ | ⋆ | ⋆ | ⋆ |  | ⋆ |  |  | 5 |
| Lee [12] | 1986 | ⋆ | ⋆ | ⋆ | ⋆ |  | ⋆ |  | ⋆ | 6 |
| Ristau [13] | 2018 | ⋆ | ⋆ | ⋆ | ⋆ |  | ⋆ |  |  | 5 |
| Wetterauer [14] | 2020 | ⋆ | ⋆ | ⋆ | ⋆ |  | ⋆ |  |  | 5 |
| John [15] | 2021 | ⋆ | ⋆ | ⋆ | ⋆ |  | ⋆ | ⋆ |  | 6 |
| Jacewicz [16] | 2021 | ⋆ | ⋆ | ⋆ | ⋆ |  | ⋆ | ⋆ | ⋆ | 7 |
| Lopez [17] | 2021 | ⋆ | ⋆ | ⋆ | ⋆ |  | ⋆ |  |  | 5 |
| Szabo [18] | 2021 | ⋆ | ⋆ | ⋆ | ⋆ |  | ⋆ | ⋆ |  | 6 |
| Bianco [19] | 2021 | ⋆ | ⋆ | ⋆ | ⋆ |  | ⋆ | ⋆ |  | 6 |
| Briggs [20] | 2021 | ⋆ | ⋆ | ⋆ | ⋆ |  | ⋆ |  |  | 5 |
| Circco-Lizza [21] | 2021 | ⋆ | ⋆ | ⋆ | ⋆ |  | ⋆ |  |  | 5 |
| Setia [22] | 2021 | ⋆ | ⋆ | ⋆ | ⋆ |  | ⋆ |  |  | 5 |
| Wertheimer [23] | 2021 | ⋆ | ⋆ | ⋆ |  |  | ⋆ |  |  | 4 |
| De Vulder [24] | 2022 | ⋆ | ⋆ | ⋆ | ⋆ |  | ⋆ |  | ⋆ | 6 |
| Ginsburg [25] | 2022 | ⋆ | ⋆ | ⋆ | ⋆ |  | ⋆ | ⋆ |  | 6 |
| He [26] | 2022 | ⋆ | ⋆ | ⋆ | ⋆ |  | ⋆ |  | ⋆ | 6 |
| Pedersen [27] | 2022 | ⋆ | ⋆ | ⋆ | ⋆ |  | ⋆ |  | ⋆ | 6 |
| Walter [28] | 2022 | ⋆ | ⋆ | ⋆ | ⋆ |  | ⋆ |  | ⋆ | 6 |
| Akinsola [29] | 2023 | ⋆ | ⋆ | ⋆ | ⋆ |  | ⋆ | ⋆ |  | 6 |
| Dhir [30] | 2023 | ⋆ | ⋆ | ⋆ | ⋆ |  | ⋆ |  |  | 5 |
| Honoré [31] | 2023 | ⋆ | ⋆ | ⋆ | ⋆ |  | ⋆ | ⋆ |  | 6 |

# 8. Evidence Profiles (Supplementary Table 6)

|  | **Certainty Assessment** | | | | | | **Number of patients** | | **Effect** | | **Certainty** | **Impor-**  **tance** |
| --- | --- | --- | --- | --- | --- | --- | --- | --- | --- | --- | --- | --- |
| **End-**  **points** | **Study design and number of studies** | **Risk of Bias** | **Incon-**  **sistency** | **Indirect-**  **ness** | **Impreci-**  **sion** | **Other considera-tions** | **Control** | **Inter-**  **vention** | **Relative OR (95%-CI)** | **Absolute** |  |  |
| **GUI** | 1 RCT (n=553)  14 NRS (n=8,155) | low risk**^o^**  high risk**^m^** | not serious**^h^** | not serious**^i^**  not serious**^i^** | serious**^j^**  serious**^j^** | not detected  not detected**^k^** | 1/277 (0.361%)  26/4,025 (0.646 %) | 3/276 (1.087%)  17/4,130 (0.412%) | 0.330 (0.034 – 3.189)  1.533 (0.807 – 2.914) | 7.26 more per 1,000  (from 6.83 fewer to 21.35 more)  2.34 fewer per 1,000 (from 14.97 fewer to 10.13 more) | **⊕⊕∅∅**  **Low^a,c^** | Important but not critical |
| **Fever** | 3 NRS (n=1,154) | high risk**^n^** | not serious**^h^** | not serious**^i^** | serious**^j^** | detected**^l^** | 8/602 (1.329%) | 6/552 (1.087%) | 0.866 (0.282 – 2.659) | 2.42 fewer per 1,000 (from 1.58 fewer to 5.39 more) | **⊕∅∅∅**  **Very low^b,d,e^** | Important but not critical |
| **Sepsis** | 7 NRS (n=3,376) | high risk**^m^** | not serious**^h^** | not serious**^i^** | serious**^j^** | not detected**^k^** | 7/1,934 (0.362%) | 6/1,442 (0.416%) | 1.303 (0.463 – 3.670) | 0.54 more per 1,000 (from 3.77 fewer to 4.86 more) | **⊕∅∅∅**  **Very low^b,d,e^** | Important but not critical |
| **Read-**  **mission** | 1 RCT (n=553)  11 NRS (n=7,270) | low risk**^o^**  high risk**^m^** | not serious**^h^** | not serious**^i^**  not serious**^i^** | serious**^j^**  serious**^j^** | not detected  not detected**^k^** | 1/277 (0.361%)  17/3,805 (0.447%) | 3/276 (1.087%)  11/3,465 (0.317%) | 0.330 (0.034 – 3.189)  1.726 (0.794 – 3.751) | 7.26 more per 1,000  (from 6.83 fewer to  21.35 more)  4.55 fewer per 1,000 (from 13.68 fewer to 4.58 more) | **⊕⊕∅∅**  **Low^a,c^** | Important but not critical |

**GRADE Working Group grades of evidence**

**High certainty:** We are very confident that the true effect lies close to that of the estimate of the effect (⊕⊕⊕⊕)

**Moderate certainty:** We are moderately confident in the effect estimate: The true effect is likely to be close to the estimate of the effect, but there is a possibility that it is substantially different (⊕⊕⊕∅)

**Low certainty:** Our confidence in the effect estimate is limited: The true effect may be substantially different from the estimate of the effect (⊕⊕∅∅)

**Very low certainty:** We have very little confidence in the effect estimate: The true effect is likely to be substantially different from the estimate of effect (⊕∅∅∅)

**Legend**: **CI,** Confidence interval; **OR,** Odds ratio; **GUI,** Genito-urinary infection; **RCT,** randomized-controlled trial, **NRS**, Non-randomized Studies

**Explanations:**

a. Downgraded by two levels for study design, as most of the studies included in the meta-analysis were retrospective in nature and judged to be unclear for sequence generation and allocation concealment

b. Downgraded by three levels for study design, as all studies included in the meta-analysis were retrospective in nature and judged to be unclear for sequence generation and allocation concealment

c. Although both RCTs examined the endpoint, only one of the RCTs could be included in the meta-analysis, as the other RCT had no events in either study arm

d. Although both RCTs examined the endpoint, neither of the RCTs could be included in the meta-analysis, as there were no events in either study arm in each case

e. Very limited inclusion of studies in the meta-analysis, as many of the studies investigating these endpoints had no events in either study arm

f. Although all 23 studies examined this endpoint, there were no events in either study arm, precluding a meta-analysis for this endpoint

h. Inconsistency was estimated as ‘not serious’ because no serious heterogeneity was detected across the NRS included (heterogeneity I^2^ = 0%)

i. The evidence is directly applicable to the question (the population, intervention and outcomes were exactly those that matched the question of the meta-analysis)

j. The 95%-confidence intervals have a medium but not a very large width, so we decided against ‘very serious’

k. Publication bias: There are no indications of publication bias, as the p-values of the Egger tests for all endpoints were >0.05 and there was no asymmetry in the funnel plots; Conflicts of interest: No conflicts of interest were identified for the authors of the study; Quality of the studies: The quality of the NRS was of course limited, but with consistent results across the studies

l. There were no indications of publication bias or conflicts of interest, but only three studies with a low level of confidence are available

m. All studies were non-randomized and evaluated using the Newcastle-Ottawa scale. No study showed a very high risk of bias, and only one study a low risk of bias. All other studies for this endpoint were attributed to a high risk of bias

n. All studies were non-randomized and evaluated using the Newcastle-Ottawa scale. All studies evaluating this endpoint had a high risk of bias.

o. The study was randomized and evaluated using the risk-of-bias tool for randomized trials (RoB 2). In each of the domains to be evaluated, a low risk of bias was observed.

# 9. Funnel plots including Egger’s tests for the different endpoints (Supplementary Figures 1a-d)

**
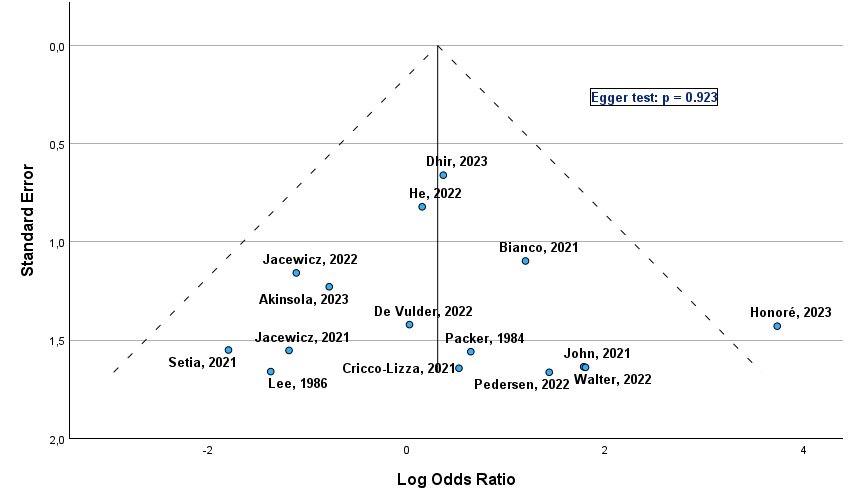
**

**Supplement Fig. 1a:** Funnel plots for publication bias in the 15 studies for the endpoint ‘rate of genitourinary tract infections following biopsy’

**
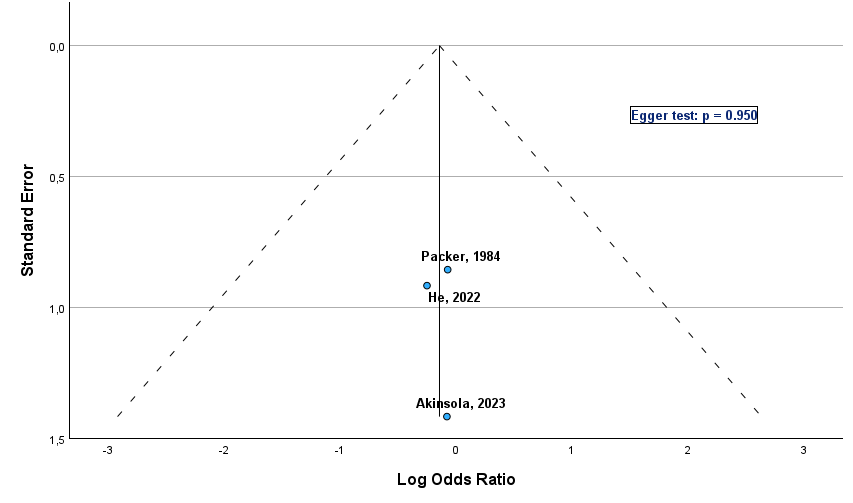
Supplement Fig. 1b:** Funnel plots for publication bias in the three studies for the endpoint ‘rate of fever following biopsy’

**
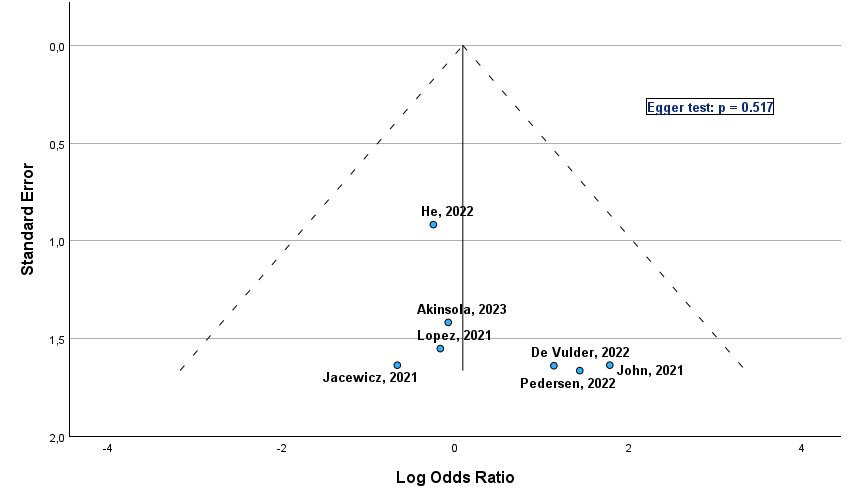
**

**Supplement Fig. 1c:** Funnel plots for publication bias in the seven studies for the endpoint ‘sepsis following biopsy’

**
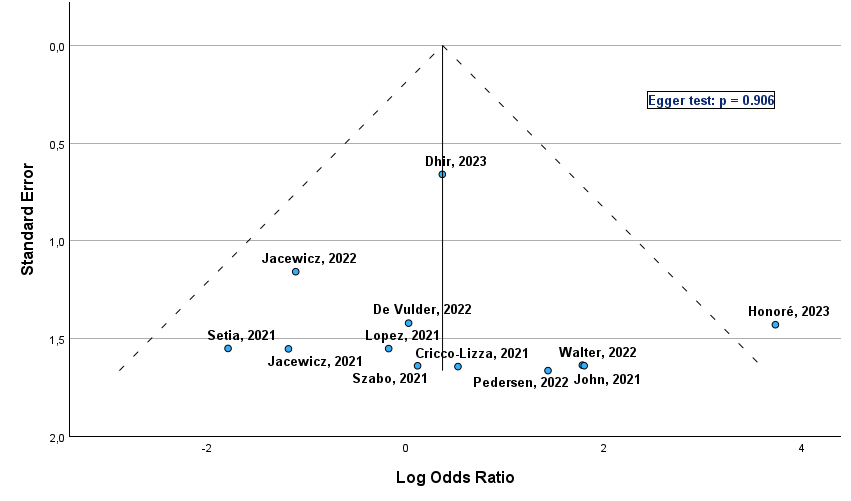
**

**Supplement Fig. 1d:** Funnel plots for publication bias in the seven studies for the endpoint ‘readmission rate for infections following biopsy’

# 10. PRISMA 2020 for Abstracts Checklist (Supplementary Table 7)

| **Section and Topic** | **Item #** | **Checklist item** | **Reported (Yes/No)** |
| --- | --- | --- | --- |
| **TITLE** | | |  |
| Title | 1 | Identify the report as a systematic review. | Yes |
| **BACKGROUND** | | |  |
| Objectives | 2 | Provide an explicit statement of the main objective(s) or question(s) the review addresses. | Yes |
| **METHODS** | | |  |
| Eligibility criteria | 3 | Specify the inclusion and exclusion criteria for the review. | Yes |
| Information sources | 4 | Specify the information sources (e.g. databases, registers) used to identify studies and the date when each was last searched. | Yes |
| Risk of bias | 5 | Specify the methods used to assess risk of bias in the included studies. | Yes |
| Synthesis of results | 6 | Specify the methods used to present and synthesise results. | Yes |
| **RESULTS** | | |  |
| Included studies | 7 | Give the total number of included studies and participants and summarise relevant characteristics of studies. | Yes |
| Synthesis of results | 8 | Present results for main outcomes, preferably indicating the number of included studies and participants for each. If meta-analysis was done, report the summary estimate and confidence/credible interval. If comparing groups, indicate the direction of the effect (i.e. which group is favoured). | Yes |
| **DISCUSSION** | | |  |
| Limitations of evidence | 9 | Provide a brief summary of the limitations of the evidence included in the review (e.g. study risk of bias, inconsistency and imprecision). | Yes |
| Interpretation | 10 | Provide a general interpretation of the results and important implications. | Yes |
| **OTHER** | | |  |
| Funding | 11 | Specify the primary source of funding for the review. | Yes |
| Registration | 12 | Provide the register name and registration number. | Yes |

# 11. PRISMA 2020 checklist (Supplementary Table 8)

| Section and Topic | Item # | Checklist item | Location where item is reported |
| --- | --- | --- | --- |
| TITLE | | |  |
| Title | 1 | Identify the report as a systematic review. | Title |
| ABSTRACT | | |  |
| Abstract | 2 | See the PRISMA 2020 for Abstracts checklist. | Suppl Table 7 |
| INTRODUCTION | | |  |
| Rationale | 3 | Describe the rationale for the review in the context of existing knowledge. | Introduction |
| Objectives | 4 | Provide an explicit statement of the objective(s) or question(s) the review addresses. | Introduction,  Suppl Table 1 |
| METHODS | | |  |
| Eligibility criteria | 5 | Specify the inclusion and exclusion criteria for the review and how studies were grouped for the syntheses. | Methods,  Suppl Table 1 |
| Information sources | 6 | Specify all databases, registers, websites, organisations, reference lists and other sources searched or consulted to identify studies. Specify the date when each source was last searched or consulted. | Methods,  Suppl Table 1 |
| Search strategy | 7 | Present the full search strategies for all databases, registers and websites, including any filters and limits used. | Methods,  Suppl Table 1, Suppl Table 2 |
| Selection process | 8 | Specify the methods used to decide whether a study met the inclusion criteria of the review, including how many reviewers screened each record and each report retrieved, whether they worked independently, and if applicable, details of automation tools used in the process. | Methods,  Suppl Table 1 |
| Data collection process | 9 | Specify the methods used to collect data from reports, including how many reviewers collected data from each report, whether they worked independently, any processes for obtaining or confirming data from study investigators, and if applicable, details of automation tools used in the process. | Methods |

| Section and Topic | Item # | Checklist item | Location where item is reported |
| --- | --- | --- | --- |
| Data items | 10a | List and define all outcomes for which data were sought. Specify whether all results that were compatible with each outcome domain in each study were sought (e.g. for all measures, time points, analyses), and if not, the methods used to decide which results to collect. | Methods |
|  | 10b | List and define all other variables for which data were sought (e.g. participant and intervention characteristics, funding sources). Describe any assumptions made about any missing or unclear information. | Methods,  Suppl Chapter 5 |
| Study risk of bias assessment | 11 | Specify the methods used to assess risk of bias in the included studies, including details of the tool(s) used, how many reviewers assessed each study and whether they worked independently, and if applicable, details of automation tools used in the process. | Methods,  Suppl Table 1 |
| Effect measures | 12 | Specify for each outcome the effect measure(s) (e.g. risk ratio, mean difference) used in the synthesis or presentation of results. | Methods,  Suppl Chapter 6 |
| Synthesis methods | 13a | Describe the processes used to decide which studies were eligible for each synthesis (e.g. tabulating the study intervention characteristics and comparing against the planned groups for each synthesis (item #5)). | Methods |
|  | 13b | Describe any methods required to prepare the data for presentation or synthesis, such as handling of missing summary statistics, or data conversions. | Methods |
|  | 13c | Describe any methods used to tabulate or visually display results of individual studies and syntheses. | Methods |
|  | 13d | Describe any methods used to synthesize results and provide a rationale for the choice(s). If meta-analysis was performed, describe the model(s), method(s) to identify the presence and extent of statistical heterogeneity, and software package(s) used. | Methods,  Suppl Chapter 6 |
|  | 13e | Describe any methods used to explore possible causes of heterogeneity among study results (e.g. subgroup analysis, meta-regression). | Methods |
|  | 13f | Describe any sensitivity analyses conducted to assess robustness of the synthesized results. | Methods |
| Reporting bias assessment | 14 | Describe any methods used to assess risk of bias due to missing results in a synthesis (arising from reporting biases). | Methods |
| Certainty assessment | 15 | Describe any methods used to assess certainty (or confidence) in the body of evidence for an outcome. | Methods |

| Section and Topic | Item # | Checklist item | Location where item is reported |
| --- | --- | --- | --- |
| RESULTS | | |  |
| Study selection | 16a | Describe the results of the search and selection process, from the number of records identified in the search to the number of studies included in the review, ideally using a flow diagram. | Results,  Fig. 1 |
|  | 16b | Cite studies that might appear to meet the inclusion criteria, but which were excluded, and explain why they were excluded. | Suppl Table 3 |
| Study characteristics | 17 | Cite each included study and present its characteristics. | Table 1 |
| Risk of bias in studies | 18 | Present assessments of risk of bias for each included study. | Results,  Suppl Table 4,  Suppl Table 5,  Suppl Fig. 1a-1d |
| Results of individual studies | 19 | For all outcomes, present, for each study: (a) summary statistics for each group (where appropriate) and (b) an effect estimate and its precision (e.g. confidence/credible interval), ideally using structured tables or plots. | Fig. 2a-2d |
| Results of syntheses | 20a | For each synthesis, briefly summarise the characteristics and risk of bias among contributing studies. | Fig. 2a-2d, Table 2, Suppl Table 6 |
|  | 20b | Present results of all statistical syntheses conducted. If meta-analysis was done, present for each the summary estimate and its precision (e.g. confidence/credible interval) and measures of statistical heterogeneity. If comparing groups, describe the direction of the effect. | Results, Fig. 2a-2d,  Table 2,  Suppl Table 6,  Suppl Fig. 1a-1d |
|  | 20c | Present results of all investigations of possible causes of heterogeneity among study results. | Suppl Table 6,  Fig. 1a-1d |
|  | 20d | Present results of all sensitivity analyses conducted to assess the robustness of the synthesized results. | Results |
| Reporting biases | 21 | Present assessments of risk of bias due to missing results (arising from reporting biases) for each synthesis assessed. | Table 2,  Suppl Table 6 |

| Section and Topic | Item # | Checklist item | Location where item is reported |
| --- | --- | --- | --- |
| Certainty of evidence | 22 | Present assessments of certainty (or confidence) in the body of evidence for each outcome assessed. | Table 2,  Suppl Table 6 |
| DISCUSSION | | |  |
| Discussion | 23a | Provide a general interpretation of the results in the context of other evidence. | Discussion,  Table 3 |
|  | 23b | Discuss any limitations of the evidence included in the review. | Discussion |
|  | 23c | Discuss any limitations of the review processes used. | Discussion |
|  | 23d | Discuss implications of the results for practice, policy, and future research. | Discussion |
| OTHER INFORMATION | | |  |
| Registration and protocol | 24a | Provide registration information for the review, including register name and registration number, or state that the review was not registered. | Methods,  Suppl Chapter 2 |
|  | 24b | Indicate where the review protocol can be accessed, or state that a protocol was not prepared. | Upon request from the corresponding author |
|  | 24c | Describe and explain any amendments to information provided at registration or in the protocol. | No amendments have been made. |
| Support | 25 | Describe sources of financial or non-financial support for the review, and the role of the funders or sponsors in the review. | The authors received no financial support for the research, authorship and/or publication of this article. |

| Section and Topic | Item # | Checklist item | Location where item is reported |
| --- | --- | --- | --- |
| Competing interests | 26 | Declare any competing interests of review authors. | Financial disclosures |
| Availability of data, code and other materials | 27 | Report which of the following are publicly available and where they can be found: template data collection forms; data extracted from included studies; data used for all analyses; analytic code; any other materials used in the review. | Upon request from the corresponding author |

# 12. References

[1] Boeve LMS, Bloemendal FT, de Bie KCC, van Haarst EP, Krul EJT, de Bruijn JJ, et al. Cancer detection and complications of transperineal prostate biopsy with antibiotics when indicated. BJU Int. 2023;132:397-403.

[2] Mian BM, Feustel PJ, Aziz A, Kaufman RP, Jr., Bernstein A, Avulova S, et al. Complications Following Transrectal and Transperineal Prostate Biopsy: Results of the ProBE-PC Randomized Clinical Trial. J Urol. 2024;211:205-13.

[3] Hughes NC, Collins PM, Power J, Soman N, Souza MD, McGarvey C, et al. The end of TRUS: Insights from a departmental transition to transperineal prostate biopsy under local anaesthesia. European Urology Open Science. 2022;43:S2.

[4] Setia S, Jackson J, Cendo D, Gorin MA, Allaway M, Vourganti S. Assessing the diagnostic performance of systematic freehand PrecisionPoint transperineal prostate biopsy: Comparison of observed outcomes to PBCG nomogram predictions. Urol Oncol. 2022;40:4 e9-4 e17.

[5] Chernysheva D, Popov SV, Orlov IN, Malevich SM, Vyazovtsev PV. The initial experience of transperineal prostate biopsy without antibiotic prophylaxis. European Urology Open Science. 2022;45:S68.

[6] Alidjanov JF, Cai T, Bartoletti R, Bonkat G, Bruyere F, Koves B, et al. The negative aftermath of prostate biopsy: prophylaxis, complications and antimicrobial stewardship: results of the global prevalence study of infections in urology 2010-2019. World J Urol. 2021;39:3423-32.

[7] Gunzel K, Magheli A, Baco E, Cash H, Heinrich S, Neubert H, et al. Infection rate and complications after 621 transperineal MRI-TRUS fusion biopsies in local anesthesia without standard antibiotic prophylaxis. World J Urol. 2021;39:3861-6.

[8] John JB, MacCormick A, MacDonagh R, Speakman MJ, Vennam R, Burns-Cox N. Complications following local anaesthetic transperineal prostate biopsies without antibiotic prophylaxis: An institution’s experience. Journal of Clinical Urology. 2022;15:385-90.

[9] Chernysheva DY, Popov SV, Orlov IN, Tsoy AV, Neraldovskiy VA. The first experience of transperineal prostate biopsy without antibiotic prophylaxis. Onkourologiya. 2021;17:46-52.

[10] Jacewicz M, Gunzel K, Rud E, Sandbaek G, Magheli A, Busch J, et al. Antibiotic prophylaxis versus no antibiotic prophylaxis in transperineal prostate biopsies (NORAPP): a randomised, open-label, non-inferiority trial. Lancet Infect Dis. 2022;22:1465-71.

[11] Packer MG, Russo P, Fair WR. Prophylactic antibiotics and Foley catheter use in transperineal needle biopsy of the prostate. J Urol. 1984;131:687-9.

[12] Lee F, Gray JM, McLeary RD, Lee F, Jr., McHugh TA, Solomon MH, et al. Prostatic evaluation by transrectal sonography: criteria for diagnosis of early carcinoma. Radiology. 1986;158:91-5.

[13] Ristau BT, Allaway M, Cendo D, Hart J, Riley J, Parousis V, et al. Free-hand transperineal prostate biopsy provides acceptable cancer detection and minimizes risk of infection: evolving experience with a 10-sector template. Urol Oncol. 2018;36:528 e15- e20.

[14] Wetterauer C, Shahin O, Federer-Gsponer JR, Keller N, Wyler S, Seifert HH, et al. Feasibility of freehand MRI/US cognitive fusion transperineal biopsy of the prostate in local anaesthesia as in-office procedure-experience with 400 patients. Prostate Cancer Prostatic Dis. 2020;23:429-34.

[15] John JB, Maccormick A, Macdonagh R, Vennam R, Speakman MJ, Burns-Cox N. Antibiotic prophylaxis is not necessary before local anaesthetic transperineal prostate biopsies - an institution’s experience of 443 consecutive cases. European Urology. 2021;79:S1406-S7.

[16] Jacewicz M, Gunzel K, Rud E, Lauritzen PM, Galtung KF, Hinz S, et al. Multicenter transperineal MRI-TRUS fusion guided outpatient clinic prostate biopsies under local anesthesia. Urol Oncol. 2021;39:432 e1- e7.

[17] Lopez JF, Campbell A, Omer A, Stroman L, Bondad J, Austin T, et al. Local anaesthetic transperineal (LATP) prostate biopsy using a probe-mounted transperineal access system: a multicentre prospective outcome analysis. BJU Int. 2021;128:311-8.

[18] Szabo RJ. Free-Hand Transperineal Prostate Biopsy Under Local Anesthesia in the Office Without Antibiotic Prophylaxis: Experience with 304 Cases. J Endourol. 2021;35:518-24.

[19] Bianco F, Luna E, Lopez-Prieto A, Shafizadeh F, Hu J, Gheiler E, et al. Prophylactic antibiotics in transperineal prostate biopsies. Are they really necessary? Journal of Urology. 2021;206:e463.

[20] Briggs LG, Kim M, Gusev A, Rumpf F, Feldman A, McGovern F, et al. Evaluation of In-Office MRI/US Fusion Transperineal Prostate Biopsy via Free-hand Device during Routine Clinical Practice. Urology. 2021;155:26-32.

[21] Cricco-Lizza E, Wilcox Vanden Berg RN, Laviana A, Pantuck M, Basourakos SP, Salami SS, et al. Comparative Effectiveness and Tolerability of Transperineal MRI-Targeted Prostate Biopsy under Local versus Sedation. Urology. 2021;155:33-8.

[22] Setia SA, Smith J, Cendo D, Yoder J, Gorin MA, Allaway MJ, et al. Outcomes of freehand transperineal prostate biopsy with omission of antibiotic prophylaxis. BJU Int. 2022;130:54-61.

[23] Wertheimer S, Budzyn J, Perkins S, Borchert A, Rogers C, Patel A. Patient Tolerability With Office Transperineal Biopsy Using a Reusable Needle Guide. Urology. 2021;154:339-41.

[24] De Vulder N, Slots C, Geldof K, Ramboer K, Dekimpe P, Uvin P, et al. Safety and efficacy of software-assisted MRI-TRUS fusion-guided transperineal prostate biopsy in an outpatient setting using local anaesthesia. Abdom Radiol (NY). 2023;48:694-703.

[25] Ginsburg KB, Drevik J, Schober JP, Bigalli AAC, Ellis JL, Braun A, et al. Antibiotic-free transperineal prostate biopsy: urology's contribution to responsible antimicrobial stewardship. Journal of Urology. 2022;207:e690.

[26] He J, Guo Z, Huang Y, Wang Z, Huang L, Li B, et al. Comparisons of efficacy and complications between transrectal and transperineal prostate biopsy with or without antibiotic prophylaxis. Urol Oncol. 2022;40:191 e9- e14.

[27] Pedersen TB, Tiessen S, Karstoft J, Poulsen MH. Introduction of free-hand MRI-guided transperineal prostate biopsies as an outpatient procedure. Dan Med J. 2022;69.

[28] Walter M, Trotsenko P, Breit HC, Keller N, Meyer A, Winkel DJ, et al. Safety profile of robotic-assisted transperineal MRI-US-fusion guided biopsy of the prostate. Front Oncol. 2022;12:1025355.

[29] Akinsola O, Bell S, Feng D, Correa A, Scarpato K. Comparing infectious complications in patients undergoing transperineal biopsy with and without antibiotic prophylaxis. Journal of Urology. 2023;209:e199.

[30] Dhir A, Zhu A, Qi J, Ferrante S, Ginsburg K, Semerjian A, et al. Is routine antibiotic prophylaxis required for transperineal prostate biopsy? Journal of Urology. 2023;209:e994.

[31] Honore A, Moen CA, Juliebo-Jones P, Reisaeter LAR, Gravdal K, Chaudhry AA, et al. Transitioning from transrectal to transperineal prostate biopsy using a freehand cognitive approach. BJU Int. 2024;133:324-31.
